# Adjunct vaginal lactic acid with single-dose metronidazole for bacterial vaginosis: a randomized controlled trial

**DOI:** 10.1186/s12905-026-04640-7

**Published:** 2026-06-30

**Authors:** Chenchit Pichailuck, Witchuda Kamolvit, Sethapong Phoonthong, Payaow Aneklap, Chanon Neungton, Perapon Nitayanon, Supathep Tansirichaiya

**Affiliations:** 1 Department of Obstetrics and Gynaecology, Faculty of Medicine Siriraj Hospital, Mahidol University, Bangkok, Thailand; 2 Department of Microbiology, Faculty of Medicine Siriraj Hospital, Mahidol University, Bangkok, 10700 Thailand; 3 Department of Nursing, Faculty of Medicine Siriraj Hospital, Mahidol University, Bangkok, Thailand

**Keywords:** Bacterial vaginosis, Lactic acid, Metronidazole, *Lactobacillus*, qPCR, Amsel criteria

## Abstract

**Background:**

Bacterial vaginosis (BV) is a common cause of vaginal dysbiosis in women worldwide, often complicated by biofilm-associated recurrence. This randomized controlled trial aimed to evaluate whether adding a 7-day course of vaginal lactic acid to a single-dose oral metronidazole improves early ecological restoration and clinical outcomes compared with metronidazole alone.

**Methods:**

An open-label, randomized controlled trial was conducted at the Department of Obstetrics and Gynaecology, Faculty of Medicine Siriraj Hospital, Mahidol University, Thailand, from December 2023 to January 2026. Women aged 18–50 years with symptomatic BV (Amsel criteria) were randomized (1:1) to receive oral metronidazole 2 g single-dose (MET) or metronidazole plus 7-day vaginal lactic acid 5 g daily (METLAC). The primary outcome was quantitative recovery of vaginal *Lactobacillus* at 10–14 days, assessed by 16S rRNA gene qPCR. Secondary outcomes included clinical cure, symptom resolution, and recurrence.

**Results:**

A total of 110 women were enrolled and randomized. Three participants were lost to follow-up, leaving 107 for the clinical analysis (MET *n* = 53; METLAC *n* = 54). Primary qPCR analysis was available for 101 participants. At 10–14 days, *Lactobacillus* 16S rRNA gene copy numbers increased significantly from baseline in both groups (*p* < 0.01). While the between-group difference in absolute *Lactobacillus* recovery was not statistically significant, the METLAC group demonstrated a numerically greater mean recovery and a higher proportion of microbiological responders (51.9% vs. 44.9%). Secondarily, at one week, the participant-reported clinical cure rate was 100% (54/54) in the METLAC group compared with 75.5% (40/53) in the MET group (absolute difference 24.5%; *p* < 0.001). Resolution of malodorous discharge was also significantly faster in the METLAC group. Recurrence rates at six months were 31.4% in the METLAC group and 21.6% in the MET group.

**Conclusions:**

Adjunct vaginal lactic acid added to single-dose oral metronidazole did not significantly improve early vaginal *Lactobacillus* recovery or reduce 6-month bacterial vaginosis recurrence. However, women receiving adjunct therapy experienced a significantly higher one-week clinical cure rate and faster malodor relief, with only mild and tolerable vaginal irritation. These findings suggest that adjunctive vaginal lactic acid provides a feasible short-term symptomatic treatment option in resource-limited Southeast Asian settings, although its role in improving long-term microbiological outcomes remains uncertain.

**Trials registration:**

Thai Clinical Trials Registry, TCTR20231106004 Registered on 6 November 2023.

**Supplementary Information:**

The online version contains supplementary material available at https://doi.org/10.1186/s12905-026-04640-7.

## Introduction

Bacterial vaginosis (BV) is a common cause of vaginal dysbiosis in women, characterized by a depletion of protective *Lactobacillus* species and an overgrowth of diverse anaerobes [1]. The condition imposes a significant burden on women’s health globally, particularly in Southeast Asia, not only due to distressing symptoms like excessive or malodorous vaginal discharge, but also due to increased risk of sexually transmitted infections, pelvic inflammatory disease and adverse pregnancy outcomes [1, 2]. In low- and middle-income settings, persistent symptoms and limited access to follow-up care compound its reproductive health impact.

The mainstay of treatment is metronidazole, which provides short-term symptomatic relief. However, recurrence occurs in more than half of affected women by 12 months [3]. The persistence of a polymicrobial biofilm on the vaginal epithelium is a major contributor to relapse and limits the effectiveness of antibiotic therapy alone [4]. Furthermore, recent microbiome studies demonstrate that short-term clinical symptom resolution alone is insufficient to predict long-term outcomes [5, 6]. Consequently, restoration of a *Lactobacillus*-dominant community is increasingly recognized as an important marker of vaginal ecological recovery and may contribute to sustained remission following treatment [7, 8]. Beyond BV, recent evidence has shown that maintaining this healthy microenvironment, including microbiota composition, local immune responses, and epithelial health, is crucial, as its disruption is linked to increased susceptibility to a broader spectrum of gynecologic disorders, such as human papillomavirus (HPV) persistence [9]. Moreover, the growing interest in localized therapeutic approaches extends beyond BV and has also been explored in other lower genital tract diseases, including HPV-associated vaginal lesions, where topical interventions have demonstrated encouraging clinical outcomes [10].

Lactic acid, the end product of glycogen in the cytoplasm of mature squamous cells, helps maintain the acidic vaginal milieu. This acidity, reinforced by *Lactobacillus* fermentation, contributes to the suppression of BV-associated anaerobes and disrupts biofilms [4, 11]. Among healthy women, common *Lactobacillus* species include *L. crispatus*, *L. gasseri*, *L. iners* and *L. jensenii* [1]. Although the lactic acid gel used as monotherapy has shown mixed results [12–14], its use as an adjunct to metronidazole may provide a complementary therapeutic strategy: metronidazole reduces bacterial load, whereas exogenous lactic acid promotes early ecological recovery. Mechanistically, exogenous lactic acid at a low pH has been shown to directly increase membrane permeability of BV-associated bacteria and down-regulate biofilm-forming genes [15, 16]. Additionally, it re-acidifies the vaginal environment, driving volatile biogenic amines (such as putrescine and cadaverine) into their protonated, non-volatile forms, thereby accelerating odor resolution [17].

Evidence supporting this approach in Southeast Asian populations remains limited. A study in Filipino women showed that adding lactic acid to a seven-day course of metronidazole resulted in a comparable cure rate but a significantly increased abundance of *Lactobacillus* from day 3 onwards [18], supporting its potential role in early ecological restoration. However, it relied on culture-based methods that underestimate fastidious or unculturable taxa, particularly *L. iners*, which may be underdetected by conventional culture-based methods [19, 20]. The efficacy of adjunctive lactic acid paired with the single-dose 2 g metronidazole regimen, a standard in Southeast Asia that ensures complete adherence but may offer less sustained antimicrobial pressure, has not yet been evaluated. A localized adjunctive treatment may enhance early microbiological recovery while maintaining the practical advantages of single-dose metronidazole therapy.

To address these gaps, we conducted a randomized controlled trial in Thailand to evaluate whether adding a 7-day course of vaginal lactic acid to a single-dose oral metronidazole improves early ecological restoration and clinical cure in women with BV. By utilizing 16S rRNA qPCR for absolute *Lactobacillus* quantification alongside Nugent scoring and complementary culture, this study aims to overcome the limitations of culture-dependent methods. This study provides molecularly informed, region-specific evidence relevant to BV management in resource-limited and high-prevalence settings.

## Methods

### Study aim, design, and setting

**Fig. 1:**
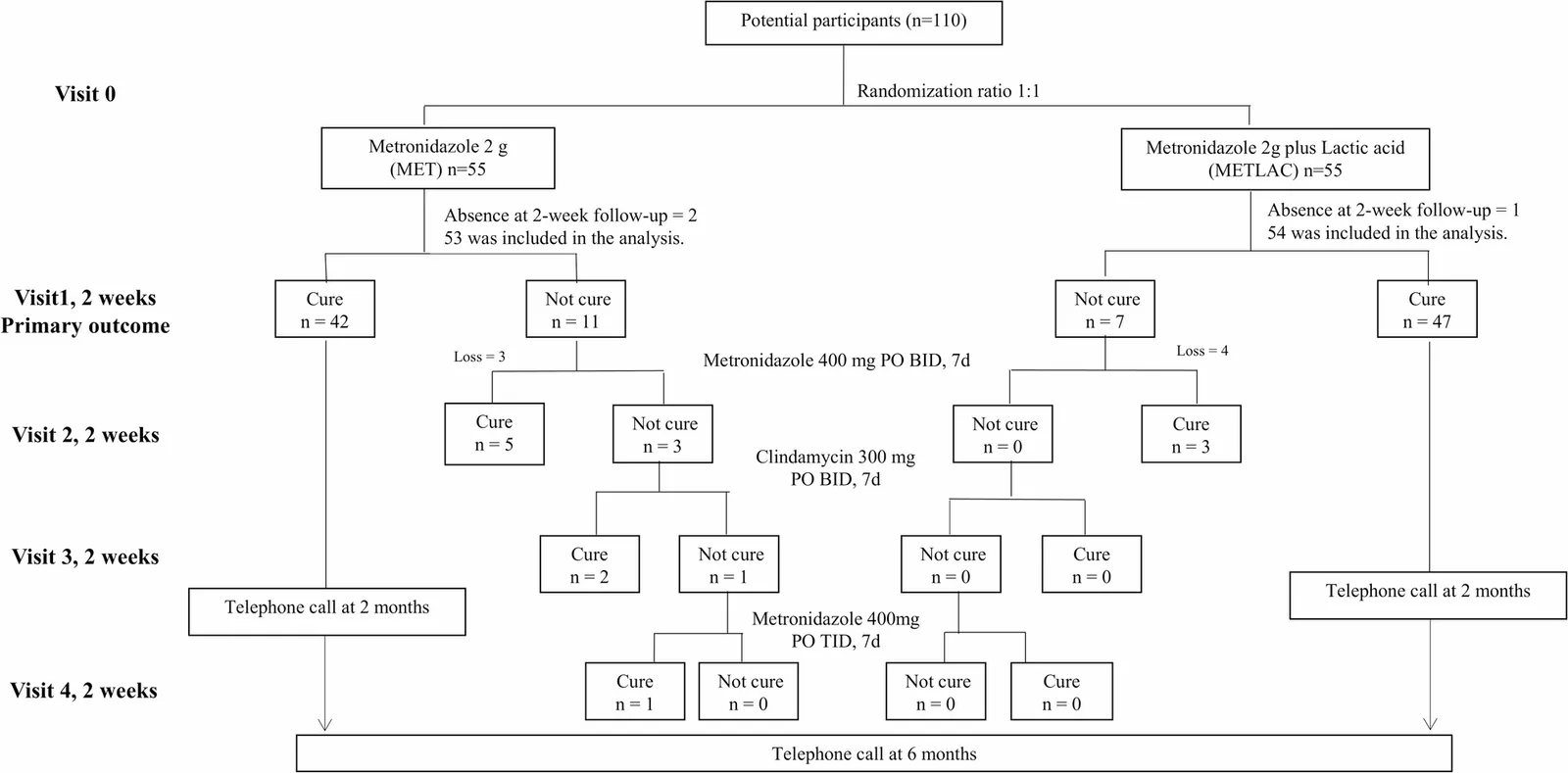
Flow of study. CONSORT flow diagram detailing the enrollment, randomization, follow-up, and analysis of study participants

This open-label, randomized controlled trial aimed to evaluate whether adding a 7-day course of vaginal lactic acid to a single-dose oral metronidazole improves early ecological restoration and clinical cure in women with bacterial vaginosis. The study was conducted at the Department of Obstetrics and Gynaecology, Faculty of Medicine Siriraj Hospital, Mahidol University, Bangkok, Thailand, between December 2023 and January 2026. Ethical approval was obtained from the Siriraj Institutional Review Board, Faculty of Medicine Siriraj Hospital (COA Si 654/2023). The study was registered with the Thai Clinical Trial Registry on 6 November 2023 before the enrolment of the first participants (TCTR20231106004). The trial adhered to CONSORT guidelines. A CONSORT flow diagram is provided as shown in Fig. 1.

Prior to enrollment, all eligible participants were informed in detail about the study, including the potential side effects of the interventions and the availability of standard-of-care treatments. Written informed consent was obtained from all participants. It was clearly communicated that participation was entirely voluntary and that declining to participate would not affect their standard medical care.

### Participants

As a routine practice, all clients presenting with abnormal vaginal discharge were asked about sexual risk behaviors and gynecological history, and underwent pelvic examination, wet preparation and pH evaluation. Amsel criteria were used to clinically diagnose bacterial vaginosis in all cases [21]. Inclusion criteria were women aged 18–50 years, presenting with abnormal vaginal discharge, a clinical diagnosis of BV, no abnormal vaginal bleeding, and absence of pregnancy. Exclusion criteria included known allergy to metronidazole or lactic acid, and any previous treatment of vaginitis in the prior two weeks. Withdrawal criteria included an invalid molecular test result and absence at the two-week follow-up.

### Randomization and masking

Participants were randomized (1:1 ratio) to receive either oral metronidazole alone (MET) or metronidazole plus vaginal lactic acid (METLAC). Randomization was performed by an independent statistician using a block-of-four, computer-generated method. Treatment allocation was kept in concealed envelopes and consecutively opened by a study nurse after participant enrollment. Due to the visible nature of the vaginal gel, participants and care providers were unblinded. However, clinical and microbiological outcome evaluators at follow-up visits remained blinded to treatment allocation.

### Interventions and procedures

Participants in the MET group received a single dose of oral metronidazole 2 g (Metrolex^®^, Siam Pharmaceutical, Thailand), consisting of five 400 mg tablets, in accordance with the Thai national guidelines, administered directly at the clinic to ensure full adherence [22]. The METLAC group received the same metronidazole dose plus 5 g of vaginal lactic acid (Canesbalance^®^, Bayer, Thailand), which consisted of lactic acid (90%), propylene glycol, methyl hydroxypropyl cellulose, sodium hydroxide and glycogen, was administered daily for seven days [23]. Participants expecting menstruation within two weeks received norethisterone acetate 5 mg (Primolut N^®^, Vinmec, Thailand) daily for 10 days to delay onset.

After one week, a research assistant conducted a clinical evaluation by telephone and also checked compliance for those assigned with lactic acid. At Visit 1 (10–14 days), blinded evaluators performed pelvic examinations and collected samples for wet preparation, Gram stain, culture, and qPCR. Participants failing to achieve clinical cure (< 3 Amsel criteria) at any follow-up visit received stepwise alternative 7-day regimens every two weeks, sequentially: oral metronidazole 400 mg twice daily (Visit 2), clindamycin 300 mg twice daily (Visit 3), and finally metronidazole 400 mg three times daily based on our previous report [24]. Recurrence was evaluated by telephone at two and six months post-treatment for those achieving clinical resolution and negative microscopic findings at Visit 1. Therefore, recurrence analyses represent a post-treatment subgroup and do not constitute a full comparison of all randomized participants.

### Clinical and demographic assessment

Demographic data, sexual health risk behaviors and personal genital hygiene were collected at baseline. Clinical evaluations at each visit assessed three symptoms (increased vaginal discharge, malodor, and itching/discomfort), graded from 0 (no) to 3 (severe). Side effects were evaluated twice: immediately at the clinic (within 30 min post-metronidazole, noting nausea/vomiting) and at one week via telephone (delayed effects from metronidazole, or local irritation from lactic acid). Clinical cure, or complete improvement of all presenting symptoms, was self-evaluated by the participants. Because participants were aware of their treatment allocation, this participant-reported outcome may have been influenced by the lack of participant blinding. Recurrence was defined as having another episode of abnormal vaginal discharge, requiring a medical visit.

### Microbiological methods

- Wet preparation and Amsel criteria

Vaginal swab was collected from the lateral and posterior fornices were examined microscopically following the Thai standard protocol to identify BV, candidiasis and trichomoniasis [22]. BV was diagnosed clinically using the Amsel criteria (requiring ≥ 3 of 4 signs: homogeneous discharge, pH > 4.5, clue cells, and positive whiff test) [21]. Resolution of bacterial vaginosis was defined as fulfillment of fewer than three Amsel criteria.

- Gram stain and Nugent scoring system

Vaginal swabs were Gram-stained and evaluated using the Nugent scoring system under a 1000x microscope (oil immersion) [25]. Scores were categorized based on the bacterial morphology, with scores ranging from 0 to 10 (0–3 = normal, 7–10 = BV) [25].

- Culture-based Detection and Identification of *Lactobacillus*

To assess viable *Lactobacillus* recovery at Visit 0 and Visit 1, swabs were streaked onto selective Lactobacillus-MRS agar and incubated at 35 °C in 5% CO₂ for 48 h. Isolates were preliminarily classified by colony morphology as *Lactobacillus* spp. or non-*Lactobacillus* organisms. Species-level identification of unconfirmed isolates was performed using matrix-assisted laser desorption/ionization time-of-flight mass spectrometry (MALDI-TOF MS) on the Bruker MALDI Biotyper system (Bruker Daltonics, Germany).

- Real-time quantitative PCR (qPCR)

Vaginal swabs were vortexed in 4.5 mL of sterile 0.85% NaCl. A 1-mL aliquot was centrifuged at 8,000×g for 5 min, and the pellet was resuspended in 20 µL pre-wash buffer (Roche, Germany), heated at 95 °C for 15 min, and briefly centrifuged [26]. Next, 180 µL of pre-wash buffer was added, followed by centrifugation at 16,300 × g for 5 min. The supernatant containing bacterial DNA was collected. The total *Lactobacillus* load was quantified by qPCR using genus-specific primers LactoF (5′-TGGAAACAGRTGCTAATACCG-3′) and LactoR (5′-GTCCATTGTGGAAGATTCCC-3′) [27]. Each 20 µL reaction contained 10 µL 2× Luna^®^ Universal qPCR Master Mix (New England Biolabs, USA), 100 nM of each primer, and 1 µL extracted DNA. Amplification on a QuantStudio 7 Pro Real-Time PCR System included 50 °C for 2 min, 95 °C for 10 min, followed by 40 cycles of 95 °C for 15 s and 64 °C for 1 min. A standard curve was generated using serial dilutions (20 fg − 44.2 ng) of *Limosilactobacillus vaginalis* genomic DNA (Accession number SAMN54807190, five 16S rRNA copies per genome). The standard curve demonstrated a strong linear relationship (R² = 0.989) with 99% amplification efficiency, enabling calculation of 16S rRNA gene copy numbers per µL. Melt-curve analysis and no-template controls confirmed amplicon specificity.

### Outcomes

The primary outcome was quantitative recovery of vaginal *Lactobacillus* at 10–14 days, assessed by changes in *Lactobacillus* 16S rRNA gene copy numbers. Absolute *Lactobacillus* quantification was selected as the primary biological marker because the restoration of a *Lactobacillus*-dominant vaginal microbiota is considered an important feature of vaginal ecological recovery following treatment for bacterial vaginosis. In addition, culture-independent molecular quantification provides greater sensitivity than conventional culture-based approaches for detecting vaginal *Lactobacillus* species. Secondary outcomes included: (1) clinical cure at one week; (2) clinical cure at 10–14 days; (3) resolution of bacterial vaginosis at 10–14 days based on Amsel criteria (< 3 criteria) [21] and (4) recurrence at two and six months, defined as the number of abnormal vaginal discharge episodes following the initial resolution at the first visit (2 weeks).

### Sample size calculation

The sample size calculation was explicitly designed to detect a difference in microbiological restoration between the two independent treatment arms. Based on foundational data showing vaginal *Lactobacillus* recovery trajectories following effective therapy [18], we hypothesized that the adjunctive lactic acid group (METLAC) would enhance *Lactobacillus* recovery compared to the metronidazole alone group (MET). To detect a medium-to-large effect size difference in the log_10_ 16S rRNA copy numbers between groups, with 80% power, a two-sided alpha of 0.05, and a 1:1 allocation ratio, 48 participants were required per arm. Allowing for 10% withdrawal or loss to follow-up, the target sample size was 55 participants per arm, giving a total of 110 participants.

### Statistical analysis

Statistical analyses were performed using STATA version 12.1, SPSS Statistics version 30.0, and GraphPad Prism version 8.0.1. Clinical and safety outcomes were evaluated for all participants who completed the follow-up assessments (*n* = 107). For the primary microbiological outcome, quantitative qPCR analysis was performed on participants with available baseline and post-treatment samples (*n* = 101), excluding 6 cases due to nonspecific amplification or primer-dimer formation. A *p* < 0.05 was considered statistically significant.

Continuous variables were compared using the Student’s t-test or Mann-Whitney U test, and categorical data using Chi-square or Fisher’s exact test. For qPCR data, log_10_-transformed 16S rRNA copy numbers were compared within groups using the Wilcoxon signed-rank test, and between groups using an independent-samples t-test for absolute change (Δ = Visit 1 - Visit 0). An analysis of covariance using a general linear model (GLM), with follow-up *Lactobacillus* copy number as the dependent variable and baseline copy number as a covariate, assessed the adjusted group effect. An exploratory responder analysis was conducted, defining a responder as achieving a ≥ 0.5 log_10_ copies/µL increase in *Lactobacillus* to capture a biologically meaningful shift; proportions were compared between groups using the chi-square test.

## Results

### Participant enrollment and baseline characteristics

From December 2023 to January 2026, 110 eligible women were enrolled and randomized. Of these, three participants (MET, *n* = 2; METLAC, *n* = 1) did not return for the two-week follow-up, leaving 107 participants available for the primary analyses (MET, *N* = 53; METLAC, *N* = 54). Participant demographic and clinical characteristics were comparable (Table 1), with the exception of a statistically significant baseline imbalance in prior malodorous discharge history, which was more frequent in the METLAC group (92.6% vs. 79.3%, *p* = 0.047). The cohort was predominantly nulliparous, non-obese, educated (with a bachelor’s degree or higher), and in their twenties or thirties. Approximately one-quarter had a history of STI, and a similar proportion reported underlying diseases. Of these participants, 77.6% were sexually active in the prior month, mostly using hormonal contraception. Excessive cleansing was common, with 85.1% always water-spraying after voiding. Abnormal vaginal discharge was prevalent in the prior year.

**Table 1:** Baseline demographic and clinical characteristics of the study participants (*N* = 107)

Characteristics	Total (*n* = 107)	MET (*n* = 53)	METLAC (*n* = 54)	*p* value
Age	32.4 ± 9.2	32.9 ± 8.2	31.9 ± 10.0	0.568
Age group				0.300
< 20	6(5.6)	1(1.9)	5(9.3)	
20–29	44(41.1)	22(41.5)	22(40.7)	
30–39	29(27.1)	17(32.1)	12(22.2)	
≥ 40	28(26.2)	13(24.5)	15(27.8)	
No of children				0.628
0	69(64.5)	32(60.4)	37(68.5)	
1	17(15.9)	10(18.9)	7(13.0)	
≥ 2	21(19.6)	11(20.7)	10(18.5)	
BMI	22.0 ± 4.2	21.3 ± 3.5	22.6 ± 4.7	0.132
BMI group				0.655
< 17	8(7.5)	5(9.4)	3(5.6)	
17–20	29(27.1)	15(28.3)	14(25.9)	
20.1–23	34(31.8)	18(34.0)	16(29.6)	
23.1–25	16(15.0)	8(15.1)	8(14.8)	
≥ 25.1	20(18.6)	7(13.2)	13(24.1)	
Education				0.616
No/primary school	2(1.9)	1(1.9)	1(1.9)	
High/vocational school	33(30.8)	14(26.4)	19(35.2)	
≥Bachelor degree	72(67.3)	38(71.7)	34(62.9)	
Sexual intercourse in the prior 1 month	83(77.6)	41(77.4)	42(77.8)	0.959
Contraception				0.982
No	27(25.2)	14(26.4)	13(24.1)	
DMPA/implant	5(4.7)	3(5.7)	2(3.7)	
COC/POP	22(20.6)	10(18.9)	12(22.2)	
IUD	5(4.7)	3(5.7)	2(3.7)	
TS	10(9.4)	5(9.4)	5(9.3)	
Others	38(35.5)	18(34.0)	20(37.0)	
No. of partners in prior 3 months	1(0–4)	1(0–4)	1(1–10)	0.470
Personal hygiene				
Water spray after voiding (always)	91(85.1)	44(83.0)	47(87.0)	0.206
Special genital soap	65(60.8)	30(56.6)	35(64.8)	0.384
Vaginal douche	16(15.0)	8(15.1)	8(14.8)	0.968
Tampon	12(11.2)	7(13.2)	5(9.3)	0.518
VC in prior 1 year	60(56.1)	28(52.8)	32(59.3)	0.503
Malodorous discharge in prior 1 year	92(86.0)	42(79.3)	50(92.6)	0.047
Detection of pseudohyphae under microscope	7(6.5)	1(1.9)	6(11.1)	0.054

*BMI * body mass index, *COC * combined oral contraceptive pill, *DMPA * depot medroxyprogesterone acetate, *POP * progestin only pill, *IUD * intrauterine device, *TS * tubal sterilization, *VC * vaginal candidiasis

### Primary outcome: quantification of *Lactobacillus* 16S rRNA copy numbers

To evaluate early ecological restoration, the quantitative recovery of *Lactobacillus* was assessed via qPCR at Visit 1 (10–14 days). The qPCR standard curve established from *L. vaginalis* genomic DNA showed a strong linear relationship (R² = 0.989) with 99% amplification efficiency (Figure S1). Of the 107 participants, qPCR data were available for 101, as six samples were excluded due to non-specific melt-curve products or primer-dimer formation. The individual Cq values and corresponding copy numbers are provided in Table S1.

**Fig. 2:**
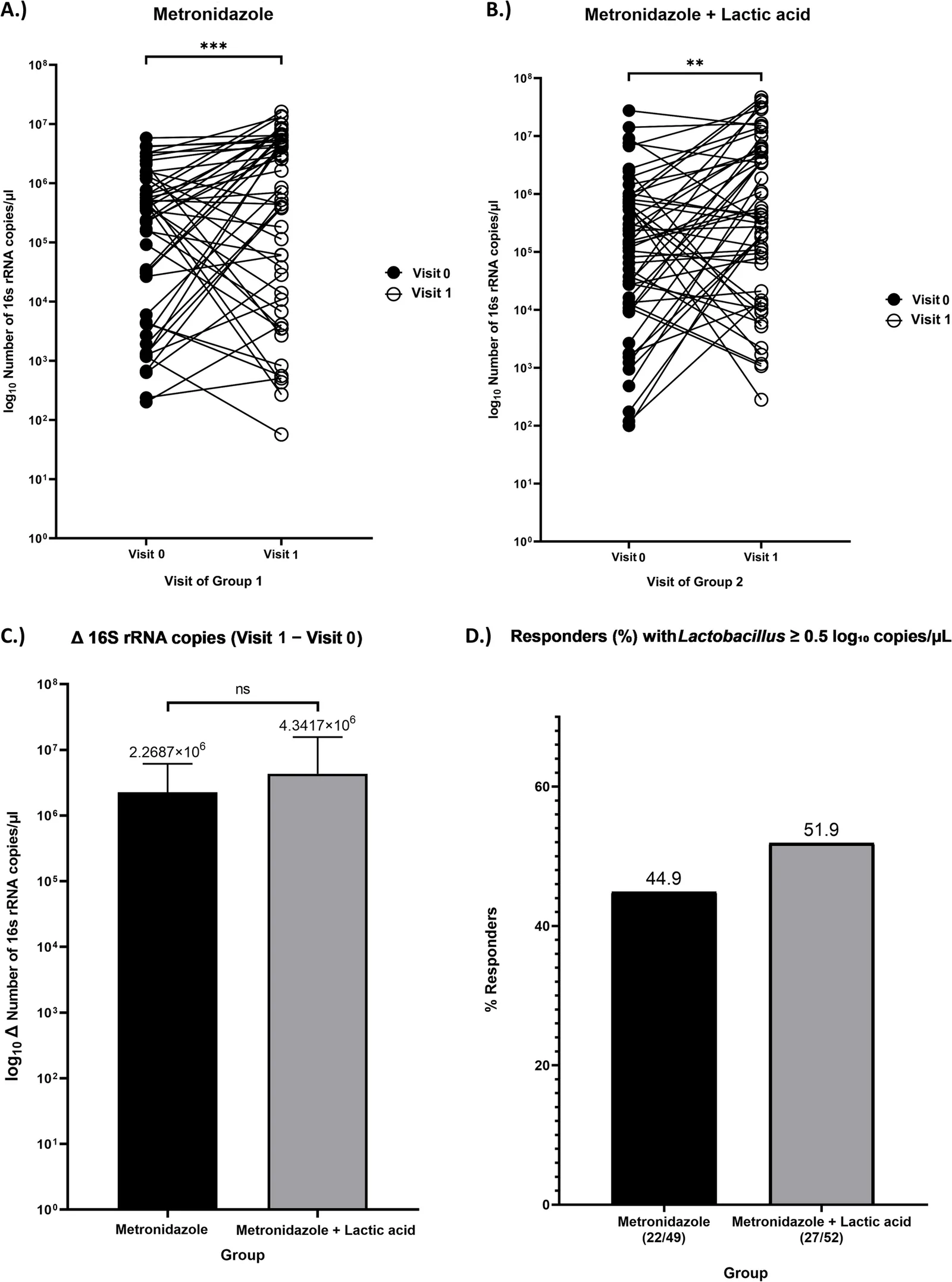
Comparison of *Lactobacillus* 16S rRNA copy numbers between visits and between groups. **A** and (**B**) Paired scatter plot showing 16S rRNA copy numbers at Visit 0 and Visit 1 in MET and METLAC, respectively. **C** Comparison of Δ values (Visit 1 - Visit 0) between MET and METLAC, presented as mean ± SEM. **D** Proportion of participants classified as responders, defined as achieving a cutoff of ≥ 0.5 log_10_ copies/µL increase in *Lactobacillus* between visits. Each line in panels (**A**) and (**B**) represents an individual participant. The y-axis shows the log_10_-transformed number of 16S rRNA copies per µL. ns = not significant; **p < 0.01; ***p < 0.001

Within-group comparisons demonstrated significant increases in *Lactobacillus* 16S rRNA copy numbers from Visit 0 to Visit 1 in both groups (Fig. 2A and B). In the MET group (*n* = 49), mean copy numbers increased from 8.55 × 10^5^ ± 1.33 × 10^6^ to 3.12 × 10^6^ ± 4.05 × 10^6^ (*p* < 0.001). Similarly, in the METLAC group (*n* = 52), values rose from 1.61 × 10^6^ ± 4.52 × 10^6^ to 5.95 × 10^6^ ± 1.14 × 10^7^ (*p* < 0.01). The GLM revealed no significant group × visit interaction (*p* > 0.05).

To compare the extent of increase in *Lactobacillus* abundance, the change in 16S rRNA copy number between visits (Visit 1 - Visit 0) was examined. The mean increase was higher in the METLAC group (4.34 × 10⁶ copies/µL) compared with MET (2.27 × 10⁶ copies/µL). This between-group difference was not statistically significant (*p* = 0.50 by independent t-test). Analysis of the log₁₀-transformed change showed a mean between-group difference of 0.22 log_10_ copies/µL (95% CI -0.43 to 0.87), with a small effect size (Cohen’s d = 0.13). This pattern is reflected in the bar graph shown in Fig. 2C, in which METLAC demonstrates a greater gain in *Lactobacillus* 16S rRNA copies.

In an exploratory analysis, responders were defined as participants achieving a cutoff of ≥ 0.5 log_10_ copies/µL increase in *Lactobacillus* between visits. The METLAC group demonstrated a numerically higher proportion of responders compared with MET (51.9% vs. 44.9%), the difference was not statistically significant (χ² = 0.26, *p* = 0.61; Fig. 2D). This trend parallels the larger mean increase observed in METLAC.

### Culture-based recovery of *Lactobacillus*

Culture-based evaluation revealed that recovery of viable *Lactobacillus* remained low overall (Table 2). When combining morphology and MALDI-TOF identifications, the MET group showed that *Lactobacillus* was recovered from 10/53 (18.9%) at baseline (Visit 0) and 11/53 (20.8%) at Visit 1. In the METLAC group, recovery increased from 10/54 (18.5%) to 12/54 (22.2%). In parallel, non-*Lactobacillus* isolates were numerically lower in both groups following treatment. The MET group declined from 32/53 (60.4%) to 25/53 (47.2%), whereas METLAC declined from 34/54 (63.0%) to 27/54 (50.0%).

**Table 2:** Culture-based bacterial recovery at baseline and Visit 1 (two weeks) in the MET and METLAC groups

Outcomes		MET group (*n* = 53)		METLAC group (*n* = 54)	
Culture	No growth	11	17	10	15
	Growth	42	36	44	39
Identification					
Morphology	*Lactobacillus*	4	5	7	6
	Non-*Lactobacillus*	27	21	30	23
MALDI-TOF	*Lactobacillus*	6	6	3	6
	- *Lactobacillus crispatus*	4	4	0	0
	- *Lactobacillus gasseri*	1	1	2	4
	- *Limosilactobacillus vaginalis*	1	1	0	1
	- *Lactobacillus delbrueckii*	0	0	1	0
	- Mixed *Lactobacillus* species	0	0	0	1
	Non-*Lactobacillus*	5	4	5	4
	- *Bifidobacterium bifidum*	1	0	3	0
	- *Candida albicans*	0	0	0	1
	- *Candida glabrata*	1	1	0	0
	- *Escherichia coli*	0	0	1	0
	- *Gardnerella*	0	0	1	0
	- *Schaalia Turicensis*	1	0	0	0
	- *Streptococcus agalactiae*	1	0	0	1
	- *Streptococcus mitis_oralis*	0	0	0	1
	- *Streptococcus salivarius*	0	0	0	1
	- *Winkia neuii*	1	0	0	0
	- Mixed non-*Lactobacillus* species	0	3	0	0
Total *Lactobacillus*		10	11	10	12
Total non-*Lactobacillus*		32	25	34	27

### Clinical outcomes and symptom resolution

**Fig. 3:**
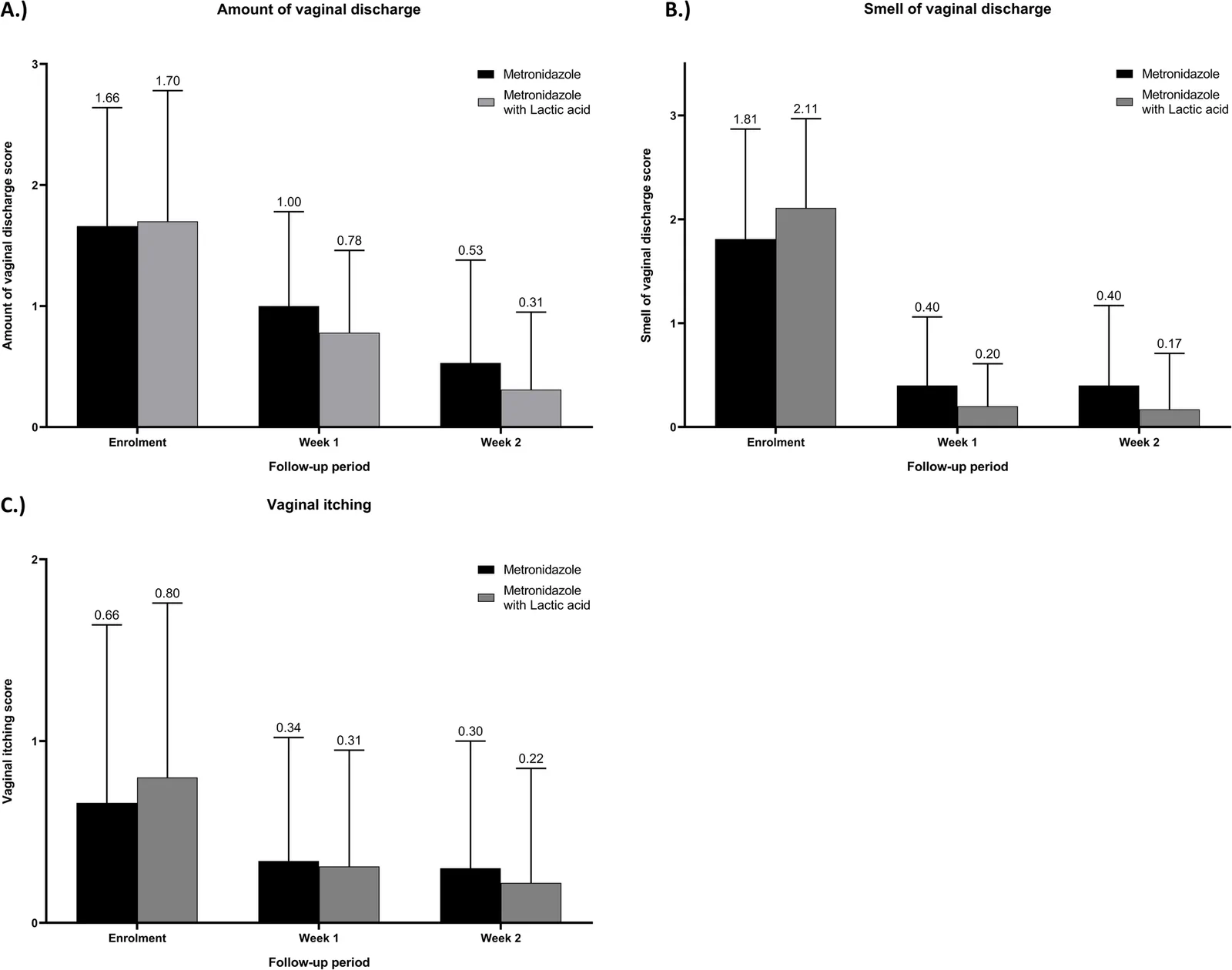
Changes in symptom scores and microbiological outcomes during follow-up. Panels **A-C** present mean symptom scores for women in MET and METLAC groups at enrolment (Visit 0), week 1 (Visit 1), and week 2. Symptoms assessed include the amount of vaginal discharge (**A**), the smell of vaginal discharge (**B**), and vaginal itching (**C**), scored using a standardized symptom scale. Bars represent mean values, with error bars indicating standard deviations

At the one-week follow-up (Visit 1), the participant-reported clinical cure rate was significantly higher in METLAC (100% vs. 75.5%; risk difference, 24.5% points [95% CI: 12.9 to 36.1]; *p* < 0.001; Table 3). However, objective Amsel-based BV resolution did not reach statistical significance (87.0% vs. 79.2%; difference, 7.8% [95% CI: -6.3% to 21.9%]; *p* = 0.282). Among those with clinical cure, time to cure was not different (METLAC 2.61 ± 1.51 vs. MET 2.52 ± 1.15 days, *p* = 0.76). Figure 3 and Table S2 show presenting symptoms at baseline and follow-up. At Visit 1, the participants in the METLAC group reported significantly less malodorous vaginal discharge. Self-rated satisfaction in both groups was high (METLAC 9.1 ± 1.2 vs. MET 8.7 ± 1.4, *p* = 0.106).

**Table 3:** Secondary clinical, microscopic, and safety outcomes at the follow-up (Visit 1)

Outcomes	Metronidazole (*n* = 53)	Metronidazole + Lactic acid (*n* = 54)	*p* value
Clinical outcomes			
Clinical cure at 1 week, *n* (%)	40/53 (75.5)	54/54 (100)	< 0.001
Side effects, *n* (%)	36/53 (67.9)	36/54 (66.7)	0.890
Self-rated satisfaction (0–10), mean ± SD	8.7 ± 1.4	9.1 ± 1.2	0.106
Days to cure	*N* = 40	*N* = 54	
Mean ± SD (95% CI)	2.52 ± 1.15 (2.16–2.89)	2.61 ± 1.51 (2.20–3.02)	0.764
Median (IQR)	3 (1.5-3)	3 (1–4)	0.962
Amsel criteria, *n* (%)			
Resolution of BV (< 3 criteria)	42/53 (79.2)	47/54 (87.0)	0.282
pH: >4.5 => ≤4.5	41/47 (87.2)	32/45 (71.1)	0.056
Clue cell: ≥20% => <20%	27/51 (47.1)	34/54 (63.0)	0.101
Whiff test: positive = > negative	43/52 (82.7)	38/45 (84.4)	0.817
Homogeneous whitish discharge: presence = > absence	43/53 (81.1)	32/43 (74.4)	0.429
Detection of pseudohyphae	9 (17.0)	10 (18.5)	0.835
Nugent score, mean ± SD			
At enrolment	7.96 ± 1.19	8.02 ± 1.46	0.687
At two weeks	4.48 ± 3.44	4.70 ± 3.39	0.687
Reduction of scores	3.48 ± 4.07	3.32 ± 3.60	0.831

At baseline, both treatment groups exhibited similarly high Nugent scores (METLAC 8.02 ± 1.46; MET 7.96 ± 1.19), with approximately 90% of participants classified as BV-positive (Table 2). Both groups demonstrated significant reductions in Nugent scores from Visit 0 to Visit 1. The MET group had a mean reduction of 3.48 points (95% CI: 2.35 to 4.61; *p* < 0.001), and the METLAC group had a mean reduction of 3.32 points (95% CI: 2.33 to 4.31; *p* < 0.001). There was no significant difference in the degree of reduction between the two groups (*p* = 0.831). This improvement was accompanied by a shift in Nugent categories, with the proportion of participants in the ‘normal’ category increasing from 0 to 16 in the MET group and from 1 to 17 in the METLAC group (Table S1). BV-positive cases decreased from 47 to 20 in MET and from 49 to 21 in METLAC. The proportion of participants demonstrating resolution of clue cells (< 20%) was higher in the METLAC group than in the MET group (63.0% vs. 47.1%, *p* = 0.101). Yeast and pseudohyphae remained low, and no trichomoniasis or gonorrhea cases were detected.

### Treatment response and long-term recurrence

At Visit 1, 18 participants did not achieve resolution of bacterial vaginosis (MET 11/53, 20.8%; METLAC 7/54, 13.0%) and received a 7-day course of metronidazole twice daily. At Visit 2, all participants in the METLAC group maintained clinical cure, whereas three in the MET group remained uncured and received a 7-day course of clindamycin. At Visit 3, one case in the MET group was still uncured and required further treatment, achieving cure by Visit 4.

Because recurrence was evaluated only in participants cured at Visit 1, this represents an exploratory subgroup analysis. At Visit 1, 42 participants in the MET group and 47 participants in the METLAC group achieved clinical resolution with negative microscopic findings and were eligible for recurrence follow-up. At two months, follow-up data were available for 42 participants in the MET group and 44 participants in the METLAC group, with recurrence rates of 11.9% (5/42) and 18.2% (8/44), respectively (difference, 6.3% [95% CI: -8.7% to 21.3%]). At six months, follow-up data were available for 37 participants in the MET group and 35 participants in the METLAC group, with recurrence rates of 21.6% (8/37) and 31.4% (11/35), respectively (difference, 9.8% [95% CI: -10.1% to 29.7%]).

### Safety and tolerability

Regarding adverse events, the only immediate side effect was nausea without vomiting (*n* = 2 in each group). The overall incidence of delayed side effects was similar between groups, with approximately two-thirds of participants reporting at least one symptom. These included nausea without vomiting (METLAC 8/54, 14.8%; MET 21/53, 39.6%), nausea with vomiting (METLAC 8/54, 14.8%; MET 7/53, 13.2%), metallic taste (METLAC 4/54, 7.4%; MET 5/53, 9.4%), dizziness (METLAC 7/54, 13.0%; MET 10/53, 18.9%), watery stool (METLAC 0/54, 0%; MET 2/53, 3.8%) and fatigue (METLAC 0/54, 0%; MET 1/53, 1.9%). Fourteen participants (25.9%) in the METLAC reported mild vaginal irritation; all participants were able to complete the full course of lactic acid.

## Discussion

To our knowledge, this is the first randomized controlled trial in Thailand to evaluate adjunct vaginal lactic acid with single-dose oral metronidazole for BV. Microbiologically, although the primary endpoint did not reach statistical significance, adjunctive therapy showed numerical trends toward greater early microbial restoration, reflected by increased *Lactobacillus* abundance and improved culture recovery. Clinically, participants in the METLAC group reported a significantly higher clinical cure rate at one week and experienced more rapid resolution of malodorous vaginal discharge, consistent with a previous study in Filipino women [18]. However, because clinical cure was participant-reported, it may have been influenced by the lack of participant blinding, and objective Amsel-based BV resolution at two weeks did not differ significantly between groups. Consistent with other studies reporting mild, tolerable vaginal irritation [15, 16, 20], side effects and time to cure were similar between groups, while recurrence was not reduced with METLAC and was numerically higher in the METLAC group at long-term follow-up.

Conventional microbiological assessments supported early treatment response. Molecular analysis provided complementary insights to the clinical findings. Although between-group differences in the primary microbiological outcome (*Lactobacillus* 16S rRNA recovery) were not statistically significant, the METLAC group exhibited consistently higher mean recovery and a greater proportion of qPCR responders. This aligns with the superior early clinical cure and malodor reduction in the METLAC arm, suggesting adjunct lactic acid may be consistent with early microbial recovery, although this was not statistically significant. While previous studies suggested that longer-term use of lactic acid might prevent recurrent episodes of BV [14, 18, 28], our short-term adjunctive regimen did not confer this long-term benefit.

qPCR was selected as the primary microbiological measure because it sensitively reflects changes in vaginal equilibrium. Unlike previous regional studies that relied on culture or microscopy [18], which often underestimate fastidious or oxygen-sensitive species such as *L. iners* [29], our use of a 16S rRNA qPCR approach provides culture-independent quantitative evidence of changes in total *Lactobacillus* abundance. This enables objective quantification of the *Lactobacillus* population regardless of viability and includes non-culturable cells within hours rather than days. The ≥ 0.5 log_10_ responder threshold reflects an exploratory and hypothesis-generating cutoff to capture a *Lactobacillus* recovery, as there is currently no universally established quantitative threshold for defining ecological cure in this context. However, total *Lactobacillus* quantification does not distinguish between individual species with distinct ecological roles [30]. In particular, protective species such as *L. crispatus* are associated with a more stable vaginal microbiota, whereas *L. iners* may represent transitional states and has been linked to a higher risk of recurrence [20, 31]. Therefore, species-specific molecular profiling or sequencing approaches may provide more biologically informative assessments of vaginal ecosystem recovery.

Culture-based results provided complementary but more conservative insights. As expected in BV, viable *Lactobacillus* recovery remained low. Nonetheless, METLAC showed a modest increase in *Lactobacillus*‑positive cultures and a numerical reduction in non‑*Lactobacillus* organisms, supporting a trend toward microbial restoration. BV‑associated taxa such as *Gardnerella*, *Schaalia*, *Streptococcus*, and *Bifidobacterium* were commonly identified at baseline, while post‑treatment isolates occasionally included transitional species (*L. gasseri* and *L. vaginalis*) consistent with early recolonization and qPCR‑defined recovery.

The observed clinical benefits are biologically plausible. Malodor in BV is primarily mediated by biogenic amines such as putrescine and cadaverine [17, 32]. Acidification by the lactic acid gel converts these volatile amines into non-volatile salts, effectively neutralizing the odor. Lactic acid is lipid-soluble and has protective effects and antimicrobial activity, including against HIV [33]. Furthermore, lactic acid attenuates mucosal inflammation by inhibiting the production of pro-inflammatory cytokines in cervicovaginal epithelial cells [34], which may explain the greater resolution of itching and irritation observed in the combination arm. The formulation used in this study contains lactic acid and glycogen [23], both of which promote resumption and growth of vaginal *Lactobacillus*, while suppressing other organisms [1]. The lactic acid concentration delivered (10 mg/mL) was nearly 10 times the natural physiological level of a healthy vagina [35], potentially contributing to its antimicrobial activity. Reported local side effects were limited to a mild degree of introital vaginal irritation, with none of the participants complaining of deep vaginal irritation. Ensuring deeper insertion of the applicator may further minimize local irritation.

The resolution rate of BV (based on Amsel criteria) in METLAC was higher than MET, which is numerically higher than the best BV regimen in our previous report, a 7-day course of metronidazole 400 mg PO TID, at 87% vs. 80% [24]. This difference may reflect the biofilm-disrupting properties of lactic acid [36]. While metronidazole effectively kills planktonic anaerobes, it often fails to eradicate the adherent *Gardnerella* biofilm on the vaginal epithelium [4, 37]. Protonated lactic acid has been shown to permeabilize the membranes of BV-associated bacteria while leaving *Lactobacillus* species intact [15, 38]. Because biofilms may re-form rapidly once lactic acid is withdrawn, maintenance or longer-term use may be required to sustain the benefit [28]. The role of lactic acid in preventing BV recurrence, as well as other biofilm-targeting approaches, such as probiotics, prebiotics and antiseptics, could be considered in future research [36].

Beyond BV alone, preservation of vaginal homeostasis is increasingly recognized as an important component of women’s health. Emerging evidence suggests that disruption of the vaginal ecosystem may influence susceptibility to recurrent infections and other lower genital tract disorders, including HPV-associated lesions [9]. In parallel, localized therapeutic approaches have been explored in a variety of gynecologic conditions, including vaginal intraepithelial neoplasia, highlighting the broader clinical relevance of strategies aimed at restoring mucosal homeostasis and maintaining a healthy vaginal environment [10]. Because chronic vaginal disorders may adversely affect quality of life and psychosocial well-being, effective and acceptable management strategies may provide benefits that extend beyond symptom resolution alone [39].

A major strength of this study is its randomized design and low loss-to-follow-up rate, ensuring the robustness of the clinical assessments. Crucially, this study fills a specific evidence gap regarding the single-dose regimen, which is often the only practical option in resource-limited settings. Adding a short course of vaginal lactic acid may provide additional short-term symptomatic relief alongside this convenient regimen while avoiding the need for extended courses of systemic antibiotics. Blinded outcome assessment minimized the possibility of measurement bias, and the use of standardized diagnostic criteria combined with quantitative molecular assessment of *Lactobacillus* by qPCR enables a rigorous and multidimensional evaluation of treatment effects. Modeling within-subject changes in 16S rRNA copy numbers provided a more precise characterization of microbiological responses than previous work.

Several limitations should be acknowledged. First, the study was open-label; participants were not blinded and were aware of their treatment assignment. Therefore, participant-reported outcomes, including short-term clinical cure and symptom improvement, may have been influenced by expectation bias or reporting bias. In addition, multiple secondary outcomes were evaluated without adjustment for multiplicity, which increases the possibility of type I error and warrants cautious interpretation of secondary findings. Furthermore, the baseline imbalance in prior malodorous discharge history (more frequent in the METLAC group) arose by chance but could theoretically have influenced the participant-reported resolution of this specific symptom. However, microbiological outcomes, including Nugent scoring and quantitative qPCR analyses, were evaluated using standardized methods by laboratory personnel blinded to treatment allocation. These objective microbiological findings showed numerical trends favoring the adjunctive lactic acid group, although between-group differences did not reach statistical significance. Second, measurement of local vaginal lactic acid concentrations was not feasible, and some participants might have difficulties with the vaginal application despite training. Third, the two‑week microbiological assessment may be too early to capture full microbiome stabilization, particularly for the slow recovery of certain *Lactobacillus* species. As a consequence, non-cured participants at Visit 1 were not included in the 2- and 6-month recurrence analyses because they received additional treatment. Finally, this single-center study in Thai women may limit generalizability; however, previous studies suggest that the predominant vaginal *Lactobacillus* species observed in Thai women are broadly comparable to those reported in other Southeast Asian populations. Previous studies have confirmed that *L. iners* is the predominant *Lactobacillus* species in healthy Thai women [40], aligning with the recovery patterns observed in our study.

Future studies incorporating high-resolution vaginal microbiome profiling could clarify species-level transition and functional pathways underlying treatment response. Integrating microbiome data with clinical outcomes could enable microbiome-informed management strategies for BV in Thai women. Improved lactic acid formulations with higher concentrations or smaller, more user‑friendly volumes may further enhance clinical effectiveness.

Overall, this study suggests that adjunct vaginal lactic acid may provide short-term clinical benefits and may support early microbiological recovery when combined with single-dose metronidazole, although the between-group difference in the primary microbiological outcome did not reach statistical significance. Given that the single-dose metronidazole regimen remains a cornerstone of BV management in many resource-limited settings due to its convenience and high compliance profile, optimizing its efficacy without resorting to extended systemic antibiotics is of high public health value. Consequently, while it does not provide a definitive microbiological advantage, this combination approach represents a potential adjunctive option for rapid symptom management in high-prevalence regions. It may offer a feasible adjunctive option for short-term symptom relief, but its effect on long-term outcomes requires further study.

## Conclusion

While both treatments significantly increased *Lactobacillus* abundance, the study did not demonstrate a statistically significant improvement in the primary microbiological endpoint or a reduction in recurrence with adjunctive vaginal lactic acid. Nonetheless, early microbial restoration was numerically greater in the combination group, though this did not reach statistical significance. Participants receiving adjunctive lactic acid reported higher short-term clinical cure rates and more rapid resolution of malodorous discharge. Side effects and time-to-cure were comparable between groups, though recurrence remains a clinical challenge. Overall, the regimen appeared feasible and generally well tolerated. Future research should explore long-term, microbiome-guided maintenance strategies to further mitigate BV recurrence.

## Supplementary Information

Supplementary Material 1.

## Data Availability

The datasets generated and/or analysed during the current study are partially included in this published article and its supplementary information files. The genomic data of the *Limosilactobacillus vaginalis* strain used for the qPCR standard curve construction are available in the NCBI BioSample database under accession number SAMN54807190. Additional participant-level clinical and microbiological data are not publicly available because they may compromise participant privacy but are available from the corresponding author on reasonable request.

## Abbreviations

BV: Bacterial vaginosis
CI: Confidence interval
Cq: Quantification cycle
DNA: Deoxyribonucleic acid
GLM: General linear model
MALDI-TOF MS: Matrix-assisted laser desorption/ionization time-of-flight mass spectrometry
MET: Metronidazole alone group
METLAC: Metronidazole plus vaginal lactic acid group
qPCR: Quantitative polymerase chain reaction
rRNA: Ribosomal ribonucleic acid
SD: Standard deviation
