# Supplementary material for: Adjunct vaginal lactic acid with single-dose metronidazole for bacterial vaginosis: a randomized controlled trial
Source: BMC Womens Health. 2026 Jun 30;26:459. doi: 10.1186/s12905-026-04640-7 (PMC13584267; doi:10.1186/s12905-026-04640-7)
Supplement: Supplementary file 1 [file 12905_2026_4640_MOESM1_ESM.docx]

**Table S1.** Individual Cq values and corresponding absolute *Lactobacillus* 16S rRNA gene copy numbers for study participants.

| **Sample no.** | **group** | **Cq Visit 0** | **Cq Visit 1** | **Number of 16s rRNA copies/μl  of Visit 0** | **Number of 16s rRNA copies/μl  of Visit 1** | **Sample no.** | **group** | **Cq Visit 0** | **Cq Visit 1** | **Number of 16s rRNA copies/μl  of Visit 0** | **Number of 16s rRNA copies/μl  of Visit 1** |
| --- | --- | --- | --- | --- | --- | --- | --- | --- | --- | --- | --- |
| 1 | MET | 19.267 | 22.866 | 1.35E+06 | 1.14E+05 | 1 | METLAC | 20.017 | 23.246 | 8.09E+05 | 4.41E+05 |
| 2 | MET | 20.778 | 20.213 | 4.80E+05 | 7.07E+05 | 2 | METLAC | 19.688 | 17.265 | 1.01E+06 | 5.35E+06 |
| 3 | MET | 20.792 | 16.925 | 4.75E+05 | 6.76E+06 | 3 | METLAC | 19.844 | 14.105 | 9.11E+05 | 4.68E+07 |
| 4 | MET | 24.751 | 17.098 | 3.14E+04 | 6.00E+06 | 4 | METLAC | 20.204 | 21.406 | 7.12E+05 | 3.12E+05 |
| 5 | MET | 18.188 | 16.986 | 2.84E+06 | 6.48E+06 | 5 | METLAC | 21.114 | 17.045 | 3.81E+05 | 6.22E+06 |
| 6 | MET | 18.008 | 15.936 | 3.21E+06 | 1.33E+07 | 6 | METLAC | 16.486 | 21.374 | 9.13E+06 | 3.19E+05 |
| 7 | MET | 17.6 | 17.356 | 4.25E+06 | 5.03E+06 | 7 | METLAC | 22.04 | 17.838 | 2.02E+05 | 3.61E+06 |
| 8 | MET | 20.081 | 15.654 | 7.74E+05 | 1.62E+07 | 8 | METLAC | 24.905 | 14.381 | 2.82E+04 | 3.87E+07 |
| 9 | MET | 20.374 | 15.904 | 6.33E+05 | 1.36E+07 | 9 | METLAC | 18.251 | 15.832 | 2.72E+06 | 1.43E+07 |
| 10 | MET | 17.629 | 17.252 | 4.17E+06 | 5.40E+06 | 10 | METLAC | 15.846 | 15.611 | 1.42E+07 | 1.66E+07 |
| 11 | MET | 19.073 | 18.17 | 1.55E+06 | 2.87E+06 | 11 | METLAC | 20.257 | 26.112 | 6.86E+05 | 1.23E+04 |
| 12 | MET | 19.441 | 24.469 | 1.20E+06 | 3.81E+04 | 12 | METLAC | 20.131 | 14.285 | 7.48E+05 | 4.14E+07 |
| 13 | MET | 24.601 | 16.38 | 3.48E+04 | 9.82E+06 | 13 | METLAC | 22.523 | 14.688 | 1.45E+05 | 3.14E+07 |
| 14 | MET | 27.666 | 30.043 | 4.24E+03 | 8.30E+02 | 14 | METLAC | 32.874 | 25.87 | 1.19E+02 | 1.46E+04 |
| 15 | MET | 20.322 | 30.982 | 6.56E+05 | 4.36E+02 | 15 | METLAC | 24.775 | 17.141 | 3.09E+04 | 5.82E+06 |
| 16 | MET | 22.313 | 26.976 | 1.67E+05 | 6.81E+03 | 16 | METLAC | 32.318 | 22.013 | 1.74E+02 | 2.06E+05 |
| 17 | MET | 18.623 | 28.337 | 2.11E+06 | 2.68E+03 | 17 | METLAC | 22.996 | 23.74 | 1.05E+05 | 6.28E+04 |
| 18 | MET | 28.327 | 17.57 | 2.70E+03 | 4.34E+06 | 18 | METLAC | 24.491 | 27.154 | 3.75E+04 | 6.03E+03 |
| 19 | MET | 31.862 | 30.761 | 2.38E+02 | 5.07E+02 | 19 | METLAC | 26.061 | 29.547 | 1.28E+04 | 1.17E+03 |
| 20 | MET | 21.006 | 25.924 | 4.10E+05 | 1.40E+04 | 20 | METLAC | 30.827 | 18.799 | 4.85E+02 | 1.87E+06 |
| 21 | MET | 24.706 | 17.659 | 3.24E+04 | 4.08E+06 | 21 | METLAC | 26.306 | 29.671 | 1.08E+04 | 1.07E+03 |
| 22 | MET | 21.837 | 18.081 | 2.32E+05 | 3.06E+06 | 22 | METLAC | 20.45 | 31.62 | 6.01E+05 | 2.81E+02 |
| 23 | MET | 19.019 | 20.89 | 1.60E+06 | 4.44E+05 | 23 | METLAC | 16.763 | 17.916 | 7.55E+06 | 3.42E+06 |
| 24 | MET | 20.812 | 27.922 | 4.69E+05 | 3.56E+03 | 24 | METLAC | 29.867 | 22.199 | 9.37E+02 | 1.81E+05 |
| 25 | MET | 23.186 | 27.944 | 9.19E+04 | 3.51E+03 | 25 | METLAC | 25.999 | 25.337 | 1.33E+04 | 2.10E+04 |
| 26 | MET | 32.109 | 27.736 | 2.01E+02 | 4.04E+03 | 26 | METLAC | 24.897 | 26.379 | 2.84E+04 | 1.03E+04 |
| 27 | MET | 29.534 | 33.949 | 1.18E+03 | 5.69E+01 | 27 | METLAC | 29.47 | 19.691 | 1.23E+03 | 1.01E+06 |
| 28 | MET | 30.438 | 24.894 | 6.33E+02 | 2.85E+04 | 28 | METLAC | 28.94 | 26.033 | 1.77E+03 | 1.30E+04 |
| 29 | MET | 20.89 | 31.688 | 4.44E+05 | 2.68E+02 | 29 | METLAC | 28.341 | 17.824 | 2.67E+03 | 3.64E+06 |
| 30 | MET | 18.424 | 17.479 | 2.41E+06 | 4.62E+06 | 30 | METLAC | 22.445 | 20.508 | 1.53E+05 | 5.78E+05 |
| 31 | MET | 17.983 | 17.676 | 3.27E+06 | 4.03E+06 | 31 | METLAC | 20.617 | 26.55 | 5.36E+05 | 9.13E+03 |
| 32 | MET | 30.35 | 20.65 | 6.72E+02 | 5.24E+05 | 32 | METLAC | 24.065 | 28.625 | 5.03E+04 | 2.20E+03 |
| 33 | MET | 22.423 | 23.772 | 1.55E+05 | 6.15E+04 | 33 | METLAC | 21.467 | 22.79 | 2.99E+05 | 1.21E+05 |
| 34 | MET | 21.247 | 22.179 | 3.48E+05 | 1.83E+05 | 34 | METLAC | 21.706 | 16.94 | 2.54E+05 | 6.69E+06 |
| 35 | MET | 29.432 | 26.301 | 1.26E+03 | 1.08E+04 | 35 | METLAC | 23.356 | 22.941 | 8.18E+04 | 1.09E+05 |
| 36 | MET | 24.822 | 16.979 | 2.99E+04 | 6.51E+06 | 36 | METLAC | 19.167 | 17.6 | 1.45E+06 | 4.25E+06 |
| 37 | MET | 17.142 | 17.003 | 5.82E+06 | 6.40E+06 | 37 | METLAC | 24.953 | 23.384 | 2.73E+04 | 8.02E+04 |
| 38 | MET | 24.994 | 23.787 | 2.66E+04 | 6.08E+04 | 38 | METLAC | 22.613 | 20.699 | 1.36E+05 | 5.07E+05 |
| 39 | MET | 27.157 | 19.959 | 6.02E+03 | 8.42E+05 | 39 | METLAC | 18.39 | 27.374 | 2.47E+06 | 5.19E+03 |
| 40 | MET | 27.593 | 30.626 | 4.46E+03 | 5.56E+02 | 40 | METLAC | 21.825 | 21.597 | 2.34E+05 | 2.73E+05 |
| 41 | MET | 29.355 | 21.113 | 1.33E+03 | 3.81E+05 | 41 | METLAC | 23.704 | 23.144 | 6.44E+04 | 9.46E+04 |
| 42 | MET | 20.672 | 20.895 | 5.16E+05 | 4.43E+05 | 42 | METLAC | 26.536 | 17.87 | 9.22E+03 | 3.53E+06 |
| 43 | MET | 29.473 | 17.279 | 1.23E+03 | 5.30E+06 | 43 | METLAC | 29.15 | 22.179 | 1.53E+03 | 1.83E+05 |
| 44 | MET | 28.822 | 21.048 | 1.92E+03 | 3.99E+05 | 44 | METLAC | 20.396 | 29.02 | 6.24E+05 | 1.68E+03 |
| 45 | MET | 20.331 | 18.331 | 6.52E+05 | 2.57E+06 | 45 | METLAC | 33.12 | 20.928 | 1.00E+02 | 4.33E+05 |
| 46 | MET | 21.91 | 17.611 | 2.21E+05 | 4.22E+06 | 46 | METLAC | 26.445 | 16.398 | 9.81E+03 | 9.70E+06 |
| 47 | MET | 21.787 | 16.581 | 2.40E+05 | 8.55E+06 | 47 | METLAC | 25.705 | 17.868 | 1.63E+04 | 3.54E+06 |
| 48 | MET | 21.126 | 16.687 | 3.78E+05 | 7.95E+06 | 48 | METLAC | 18.739 | 16.102 | 1.94E+06 | 1.19E+07 |
| 49 | MET | 20.523 | 18.984 | 5.72E+05 | 1.64E+06 | 49 | METLAC | 19.678 | 21.087 | 1.02E+06 | 3.88E+05 |
|  |  |  |  |  |  | 50 | METLAC | 22.837 | 19.57 | 1.17E+05 | 1.10E+06 |
|  |  |  |  |  |  | 51 | METLAC | 14.875 | 15.75 | 2.76E+07 | 1.51E+07 |
|  |  |  |  |  |  | 52 | METLAC | 16.924 | 14.786 | 6.76E+06 | 2.93E+07 |

**Table S2.** Nugent scores and microscopic findings at baseline and two weeks after treatment.

| **Variables** | **MET group (n=52)^*^** | | **METLAC group (n=54)** | |
| --- | --- | --- | --- | --- |
|  | **Visit 0** | **Visit 1** | **Visit 0** | **Visit 1** |
| **Nugent Score** | 7.96 ± 0.16 | 4.48 ± 0.48 | 8.02 ± 0.20 | 4.70 ± 0.47 |
| **Normal (0-3)** | 0 | 16 | 1 | 17 |
| **Intermediate (4-6)** | 5 | 16 | 4 | 16 |
| **BV Positive (7-10)** | 47 | 20 | 49 | 21 |
| Reduction of scores  (Visit1 – Visit2) | 3.48 ± 0.56 | | 3.32 ± 0.50 | |
| **%Clue cell** | 17.82 | 4.25 | 16.94 | 3.80 |
| **Pseudohyphae** | 3 | 4 | 3 | 4 |
| **Yeast** | 6 | 8 | 8 | 10 |
| ***Trichomonas*** | 0 | 0 | 0 | 0 |
| ***N. gonorrhoeae*** | 0 | 0 | 0 | 0 |
| **White Blood Cell** | 19.60 | 26.37 | 16.35 | 23.11 |

^*^One sample in the MET group had missing Nugent score data at Visit 0; therefore, analyses for this group were conducted using N=52 for both visits.

**Figure S1.** Standard curve for the quantitative PCR assay. The standard curve was established using a 10-fold serial dilution of *Lactobacillus* *vaginalis* genomic DNA, demonstrating a strong linear relationship (R^2^ = 0.989) with an amplification efficiency of 99%.

Standard Curve for Absolute Quantification of 16S rRNA Gene

| Cq | log10 Number of 16s rRNA copies/μlitle |
| --- | --- |
| 13.97 | 8.03768 |
| 14.048 | 7.94077 |
| 14.069 | 7.81583 |
| 14.238 | 7.63974 |
| 14.826 | 7.33871 |
| 15.635 | 6.9186 |
| 17.689 | 6.23611 |
| 21.467 | 5.39226 |
| 27.545 | 3.69329 |
| 34.498 | 1.69329 |
